# Robotic partial cystectomy with excision of mesh after inguinal hernia repair: a case report

**DOI:** 10.1186/s12894-023-01197-7

**Published:** 2023-02-28

**Authors:** Christian Ramesmayer, Lukas Lusuardi, Hubert Griessner, Ricarda Gruber, Lukas Oberhammer

**Affiliations:** 1 Department of Urology and Andrology, Paracelsus Medical University, Müllner Hauptstraße 48, 5020 Salzburg, Austria; 2 Department of Urology and Andrology, Pyhrn-Eisenwurzen Klinikum Steyr, Steyr, Austria

**Keywords:** Mesh erosion, Inguinal hernioplasty, Case report, Calculi, Robotic

## Abstract

**Background:**

Mesh erosion into the bladder after hernioplasty is sparsely reported in literature and may be underestimated in clinical practice. We report a case of a patient who was referred to our department due to recurrent urinary tract infections caused by a bladder stone due to mesh migration after inguinal hernia repair 22 years ago.

**Case presentation:**

A 67-year-old male patient was referred from the outpatient urologist for transurethral resection of the prostate in September 2021 due to recurrent urinary tract infections caused by benign prostatic enlargement and bladder stone formation. During the operation, parts of the stone were smashed and the prostate was resected. Additionally, a mesh eroding from the bladder roof was detected masqueraded by the stone. A computed tomography scan, which was performed afterwards, revealed a 20 × 25 mm mesh migration into the bladder after inguinal hernia repair on the left with concomitant stone adhesion to the mesh. After revealing patient history, an inguinal hernia repair with mesh implantation was done 22 years ago. A robotic assisted partial cystectomy and mesh excision was performed. The patient recovered well.

**Conclusion:**

Mesh erosion into the urinary bladder after hernia repair can occur up to two decades after the primary operation. Although it is rarely reported, it can be a possible cause for recurrent urinary tract infections and therefore a mentionable complication after inguinal hernia operation. Robotic-assisted laparoscopic partial cystectomy with complete excision of the mesh is an option for definitive treatment.

## Background

Urinary calculi formation in the bladder is a common cause for urinary tract infection (UTI). These stones are often found in prostate hyperplasia, the main etiology for lower urinary tract obstructive diseases. Another important cause leading to bladder stone formation are foreign bodies. In literature, there were only few case reports about mesh erosions into the bladder causing urinary stones and concomitant recurrent UTI. The rising count of hernia repair procedures with prosthetic mesh will probably increase the number of severe complications such as mesh erosions. We are reporting a rare case of urinary bladder stone formation due to mesh migration after hernioplasty, which was performed 22 years ago.

## Case presentation

**Fig. 1:**
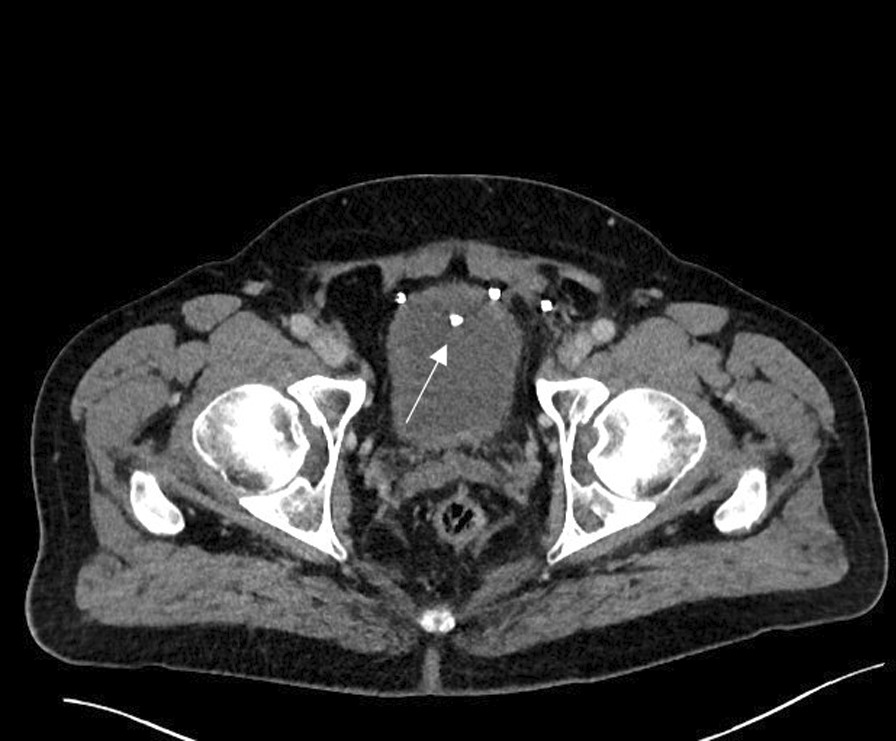
Computed tomography scan showing a stone in the bladder roof indicated by white arrow

A 67-year-old male patient was referred from the outpatient urologist for transurethral resection of the prostate (TUR-P) in September 2021 due to recurrent UTI. After treating the patient with several antibiotics, the urologist performed a cystoscopy, which revealed a bladder stone and an obstructive prostate. Finally, the patient was referred to our department for TUR-P. The initial hypothesis was, that the obstructive prostate caused the bladder stone. During the operation, parts of the stone were smashed and the prostate was resected. Furthermore, a prosthetic mesh eroding from the bladder roof was detected. The mesh was masqueraded by the stone. The pathological result ruled out any malignancy. A postoperative computed tomography scan revealed a 20 × 25 mm mesh migration into the bladder after inguinal hernia repair on the left (Fig. 1) with concomitant stone adhesion to the mesh. After revealing patient history, the inguinal hernia repair was performed 22 years ago.

**Fig. 2:**
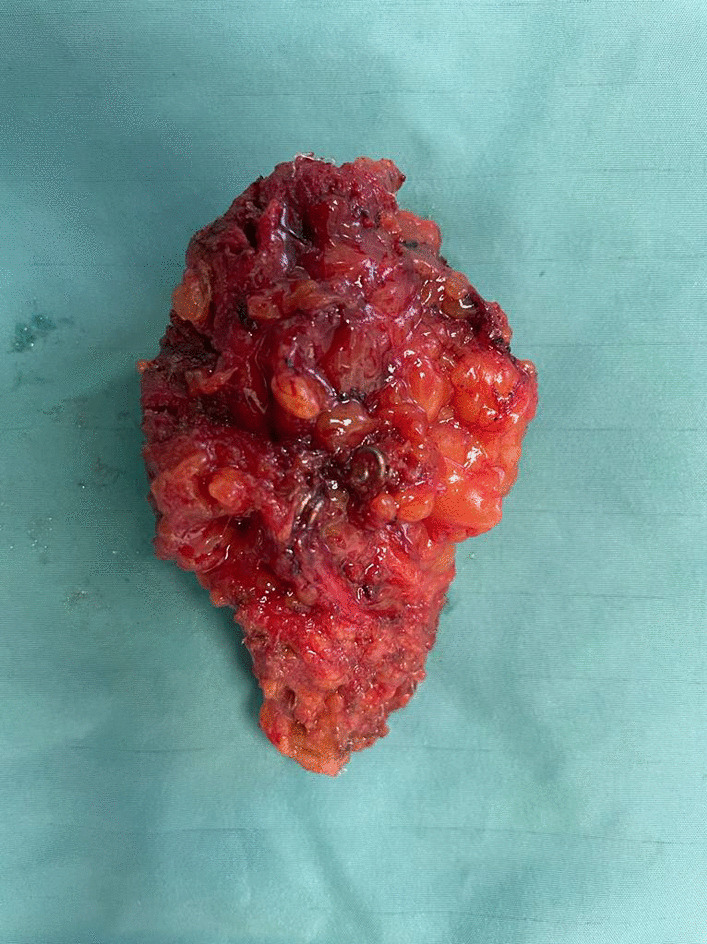
Resected mesh and stone after robotic assisted laparoscopic partial cystectomy

**Fig. 3:**
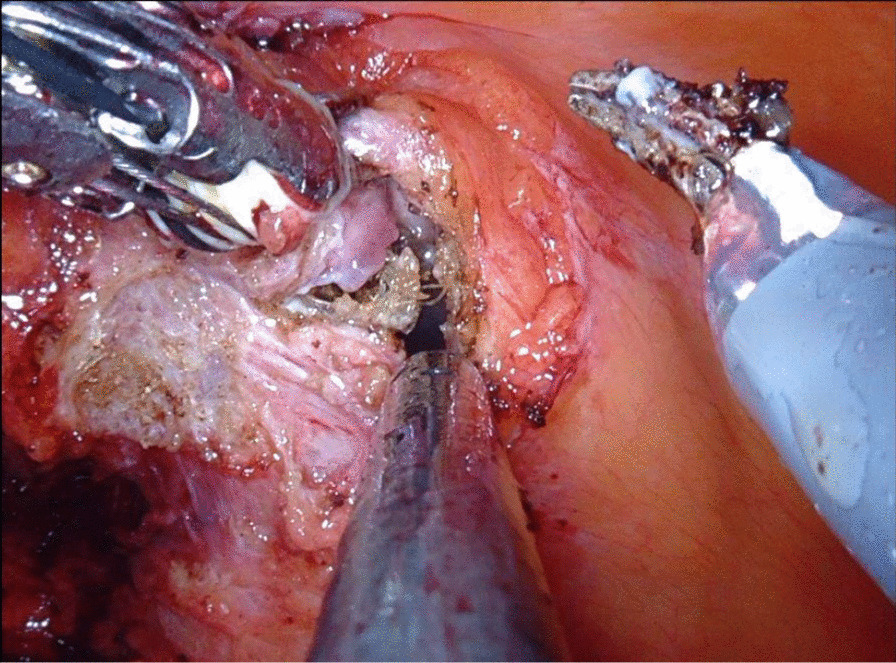
Intraoperative picture showing mesh after hernioplasty eroding into the bladder

The patient was admitted to robotic-assisted laparoscopic partial cystectomy with excision of the complete mesh and the stone (Fig. 2, and  3). The procedure was performed using the “da Vinci X Surgical System” (Intuitive Surgical, Sunnyvale, CA). The patient was placed in Trendelenburg position. The operation was performed transperitoneal with a 12-mm camera port supraumbilical and three 8-mm trocars on a straight line. Additionally, one 8-mm port for the assistance was placed on the right. After partial resection of the bladder, the defect was closed with two-layer V-loc sutures (3.0). Lapra-Ty clips were attached at each suture for safety issues. Next a transurethral catheter was placed. No drainage was necessary due to the treating surgeon’s decision. The patient recovered well. After a normal cystography on postoperative day 7, the foley catheter was removed. The final pathological results revealed chronic fibrosis and mucosa inflammation of the bladder. After almost one year of follow-up the patient has no symptoms regarding hernia or voiding.

## Discussion

The etiologies of urinary tract infection are heterogeneous. Benign prostatic enlargement and following bladder stone formation is the most frequent cause. Foreign bodies eroding into the bladder causing stones is rarely reported in literature: In 2019, Li and Cheng made a systematic research and described 23 cases of mesh erosion after hernia repair since 1994 [1]. The most frequently reported hernia repair surgery was the TAPP technique.

The reasons for mesh erosions are unclear. Inflammatory responses to the viscera due to the sharp edges of the mesh could be a possible explanation according to Chowbey et al. [2]. Mechanical migration due to external forces or induced by foreign body reactions are other theories, which were published by Agarwal et al. [3].

In literature, the period from primary hernia repair and urological complications as mesh erosion for instance, ranged from 3 months to 20 years. Interestingly, almost 50% of the reported cases had a latency of over five years until complications occurred. Furthermore, one patient presented with a mesh erosion after 20 years after surgery [4].

When reviewing literature, there were only three reports about laparoscopic management of mesh migration into the bladder [5–7]. There is only one reported case of robotic excision of the mesh [8].

Computed tomography scan and cystoscopy are important tools in these cases. After transurethral resection, we were able to detect the real etiology of the UTI. The initial cystoscopy could not detect the migrating mesh, which was masqueraded by the stone.

For definitive treatment partial cystectomy with complete removal of the mesh and bladder repair is recommended [2, 9, 10]. Even though the open approach is most frequently reported, laparoscopic and especially robotic surgeries with their advantages of faster recovery, better visualization and shorter hospital stay, may be surgical techniques, which should be preferred in the future.

This case report underlines the importance of further differential diagnosis when uncommon bladder stones on the bladder wall are detected and if the patient has a history for mesh-hernia repair.

## Conclusion

Although only a few case series about mesh migration into the bladder with concomitant calculi formation after hernioplasty have been published, it should be considered as rare but possible cause of lower urinary tract infections. Robotic assisted partial cystectomy with stone removal may be an option for definitive treatment.

## Data Availability

All data generated or analysed during this study are included in this published article.

## Abbreviations

TUR-P: Transurethral resection of the prostate
UTI: Urinary tract infection
